# Lip Augmentation With Saypha LIPS Lidocaine: A Postmarket, Prospective, Open-Label, Randomized Clinical Study To Evaluate Its Efficacy and Short- and Long-term Safety

**DOI:** 10.1093/asj/sjae149

**Published:** 2024-08-21

**Authors:** Daniel S Müller, Doris Grablowitz, Alice Krames-Juerss, Artur Worseg

## Abstract

**Background:**

Versatility, biocompatibility, and reversibility make hyaluronic acid fillers the backbone of minimally invasive lip augmentation procedures.

**Objectives:**

The aim of this study was to assess the effectiveness and short- and long-term safety of Saypha LIPS Lidocaine (Croma Pharma, Leobendorf, Austria) for lip augmentation to correct moderate to severe lip volume deficiency (grade 1-3 lip fullness score [LFS]).

**Methods:**

In this postmarket, prospective, open-label, multicenter, randomized clinical study, 114 patients were initially treated (with optional touch-up treatment at Week 3). Retrograde and bolus techniques were employed with defined needles or cannula. The primary effectiveness endpoint was the proportion of patients with lip volume improvement of LFS ≥ 1 grade vs baseline at Week 6 (ie, responders); with follow-up (FU) for secondary effectiveness at Months 6, 12, and 18. Evaluation scores included the LFS, Global Aesthetic Improvement Scale (investigator and patient), patient satisfaction questionnaire FACE-Q, and a numerical pain rating scale.

**Results:**

At Week 6, >90% of the patients were responders (lower-lip: 95% CI, 92.24-99.43, *P* = .0071; upper-lip: 95% CI, 90.95-99.00, *P* = .0234), with post hoc analyses showing the outcome was influenced by the initial volume deficiency and total volume injected, but not by touch-up treatment. At Month 6, 90% of the patients were responders; at Month 12, 70%; and at Month 18, >40% still had a visible effect. Adverse events were mostly procedural, mild, and temporary. Pain perception was significantly reduced 15 minutes after the procedure. Aesthetic improvement and patient satisfaction were rated as high at all time points.

**Conclusions:**

Saypha LIPS Lidocaine for lip augmentation showed long-term aesthetic improvement and safety.

**Level of Evidence: 2:**

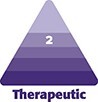

For the past 25 years, the popularity of soft tissue fillers has increased substantially, and this minimally invasive procedure is now commonly requested by patients as they look for a more youthful appearance, or to alter certain features of their face that they find undesirable.^1,2^

Although other options exist, hyaluronic acid (HA) fillers have emerged as the primary choice for minimally invasive soft tissue augmentation, especially to enhance the lips, due to this product's versatility, biocompatibility, and reversibility with hyaluronidase.^1,3,4^ There is a high demand for lip augmentation procedures with HA in contemporary clinical practice.^1^

Saypha LIPS Lidocaine (SLL; Croma-Pharma GmbH, Leobendorf, Austria) is a sterile, biodegradable, viscoelastic, clear, absorbable, isotonic, and homogenized injectable HA gel implant approved for use in the European Union. It contains HA at a concentration of 23 mg/mL in a physiological buffer and is cross-linked with 1,4-butanediol diglycidyl ether (BDDE), which covalently binds native HA molecules to each other with repeating bridges. The formulation of the HA gel implant includes lidocaine hydrochloride (0.3% weight/weight), a supplemental anesthetic intended to bypass the need for additional anesthesia during the injection procedure. Lidocaine provides a numbing effect, ensuring patient comfort during the treatment.^5^ This not only improves overall patient satisfaction by minimizing pain, but also enables a more precise, swift, and effective treatment, reducing procedural time through a streamlined approach combining anesthesia and treatment in a single step.^6^

SLL is a gel implant with a low G′ (elastic modulus) of approximately 150,000 mPa (at 1 rad/s) and a G′ (viscous modulus) of approximately 27,000 mPa, in which the elasticity is more pronounced than the viscosity. These values highlight a filler with much more elastic than viscous properties, enabling it to maintain its shape against the dynamic forces of lip movement.

HA products with a lower G′ are ideal for treating the lip area because they can be placed in a superficial plane, and result in volume enhancement without distortion of the natural motion of this highly dynamic facial region.^1^ Therefore SLL is indicated for lip augmentation.^7^

Although HA filler treatments entail various risks, adverse events (AEs) are generally mild, self-limiting, and, importantly, reversible.^8^ However, it remains critically important to thoroughly comprehend the product, including its properties and associated risks, and to ensure predictability regarding the treatment outcome.^9^ Therefore, it is imperative that the safety and effectiveness of HA implants continue to be monitored during routine clinical practice after market approval.^10^ In this study we assessed the effectiveness and the short- and long-term safety of SLL to correct moderate to severe lip volume deficiency.

## METHODS

### Study Design

This was a postmarket, prospective, open-label, multicenter, 1:1 randomized, noncomparative clinical study conducted from July 20, 2020, to April 6, 2022, at 3 independent Austrian sites coordinated by 3 investigators. Simple 1:1 randomization was done digitally by an electronic case report form (eCRF) system. The Ethics Committee approval was obtained from the Ethikkommission Medizinische Universität Wien (Ethics Committee of Medical University of Vienna, Austria) and Ethikkommission der Stadt Wien (Ethics Committee of the City of Vienna, Austria); and the study was registered at ClinicalTrials.gov (NCT04917588). The investigation was conducted in accordance with International Standards Organization ISO14155, the principles of the Declaration of Helsinki, and the applicable sections of the respective national medical device laws.

The primary objective of this clinical investigation was to assess the effectiveness and the short- and long-term safety of SLL for lip augmentation for a total period of 18 months. On Day 0 (Visit 1) patients underwent the treatment, with a possible touch-up treatment at Week 3 (Visit 2), if the investigator deemed the correction was not optimal after the first injection. A telephone visit was performed 24 hours after each treatment for the safety evaluation (Visit 1a and Visit 2a in case of touch-up treatment). Patients then returned for follow-up (FU) assessments at Week 6 (Visit 3), Month 6 (Visit 4), Month 12 (Visit 5), and Month 18 (Visit 6). If it was observed by the investigator that no aesthetic effect was visible after 12 months, the study could be terminated for the respective participant.

### Study Cohort

**Figure 1.**
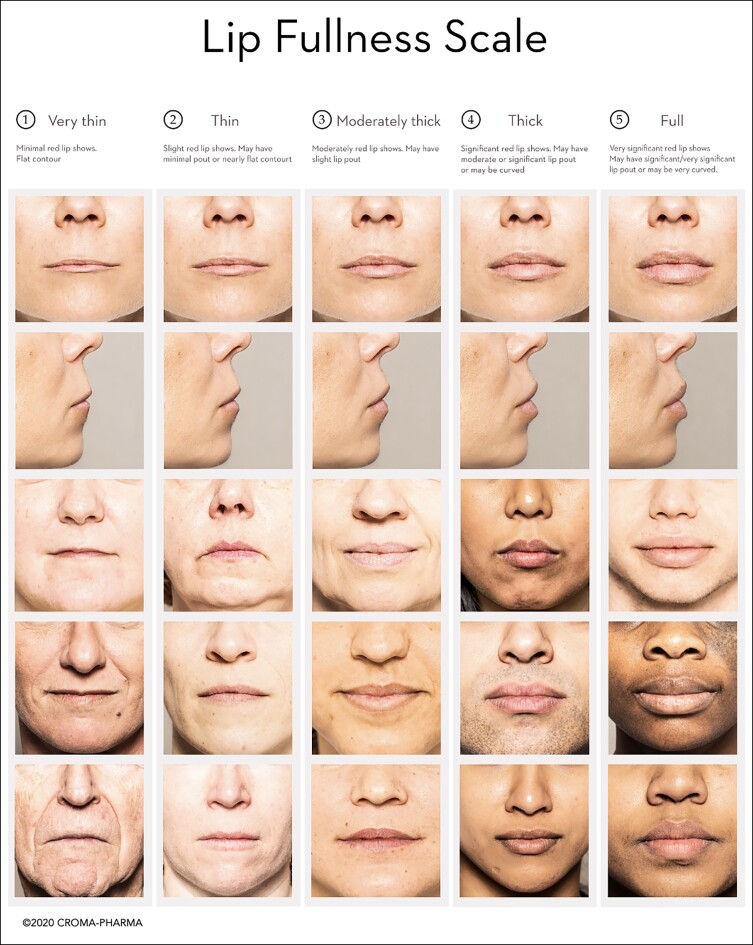
Lip fullness scale (LFS) descriptors and associated reference images.

Eligible participants were males and females (≥18 years old) with the presence of approximately symmetrical “very thin” lips (minimal red lip shows) to “moderately thick” lips (moderate red lip shows) as assessed with the lip fullness scale (LFS), a validated 5-point photonumeric rating scale that objectively quantifies fullness of the upper and lower lips separately (inclusion of severity grade of 1 to 3 on both lips; Figure 1) and determined by the investigator at Visit 1 (live assessment). The LFS was previously independently validated for clinical use by determining its interrater and intrarater reliability. Interrater agreement was found to be “substantial” to “almost perfect,” with intraclass correlation (ICC) values ranging from 0.80 to 0.98 for photographs and 0.83 to 0.96 for live assessments. Intrarater agreement was also “moderate” to “almost perfect,” with kappa values between 0.64 and 0.91 and ICC values between 0.94 and 0.98. Overall, the Croma Lip Fullness Scale demonstrated high reliability, assessed as “substantial” to “almost perfect” by expert reviewers (further description is found in the Appendix, located online at www.aestheticsurgeryjournal.com). Furthermore, eligible participants had healthy facial skin, and were willing to abstain from any aesthetic or surgical procedures in the treatment area for the duration of the clinical investigation. Exclusion criteria are specified in the supplemental file, located online at www.aestheticsurgeryjournal.com. Before any investigation-related procedures or assessments were performed, patients voluntarily signed the informed consent form, with the option to withdraw and leave the investigation at any time (final assessment was made at the time of withdrawal).

### Treatments

**Figure 2.**
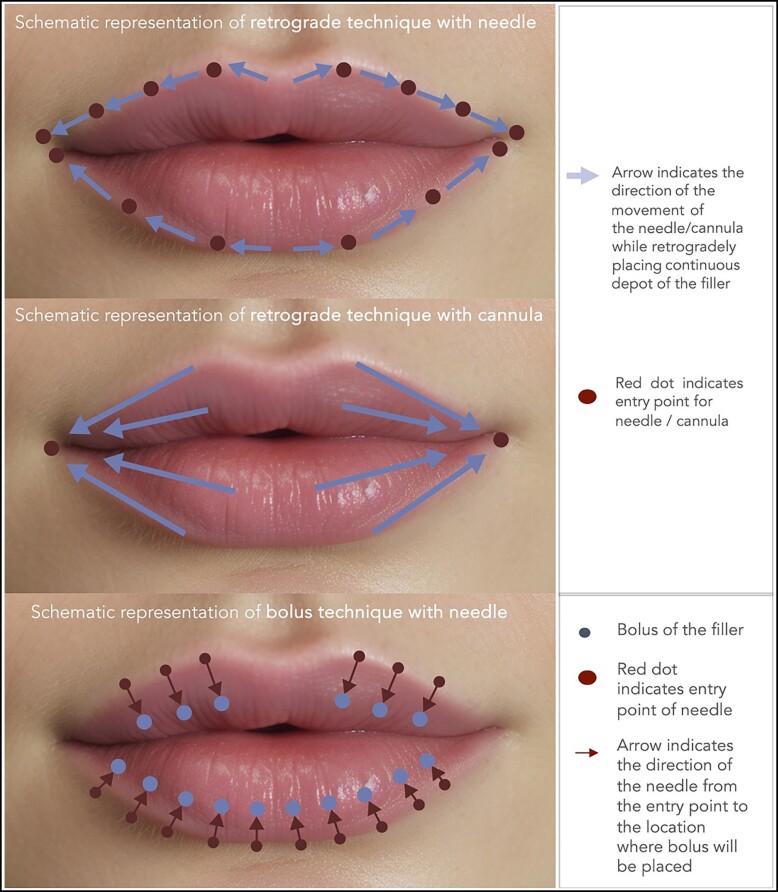
Lip application techniques: retrograde and bolus.

SLL was administered to eligible participants on Day 0, with either the retrograde or the bolus technique, which could be injected with a choice of a 27-gauge 1/2-inch needle or 25-gauge × 50-mm/22-gauge × 70-mm cannulas, based on the characteristics of the patient's defect and the investigator's preference. Detailed instructions regarding injection techniques were provided to the investigators (Figure 2); and the performance of the treatment was analyzed separately for each patient subgroup: (1) retrograde, (2) bolus, (3) needle, and (4) cannula. Briefly, for the retrograde technique with needle and cannula, investigators introduced the entire needle/cannula along the upper and lower lip, respectively, and injected the product in the submucosa, while slowly withdrawing the needle/cannula. For the bolus technique with needle, there was deposition of a small bolus just before withdrawing the needle. The bolus injections were placed subdermally from the white portion of the lip directly into the mucosa. If a larger volume of filler was needed to achieve the correction, small volumes were injected in 2 sessions, instead of a higher volume in 1 session. The device was to be injected slowly, with the least amount of pressure. Aspiration was conducted before injection to verify that the needle was not intravascular. The injection was stopped just before the needle/cannula was pulled out of the skin to prevent leakage of the device or placement too superficially in the skin.

An optional touch-up treatment was done at Week 3 (Visit 2), if according to the investigator optimal results were not achieved with the initial treatment. The volume applied depended on the size of the area to be treated and the severity of volume deficit. The maximum recommended volume of SLL was 10 mL per treatment session (both lips included); and a total of 20 mL per participant per year, with a total FU of 18 months.

### Effectiveness Endpoints

On Day 0 (before treatment) and at all FU visits except the Week 3 visit, photographs of the participants lips were taken by the investigator with a 3-dimensional (3D) digital camera system (LifeViz Mini 3D Imaging System; Quantificare S.A., France). A standardized procedure was applied to ensure consistency for each participant and between the visits and across sites. All photographs were reviewed and graded by the investigator and participants for GAIS assessment; and, in random order, by an independent photography reviewer, who was a dermatologist with an aesthetic medicine focus, utilizing the LFS grading system described in Figure 1 for all FU visits. The review by an independent photographer was intended to allow for central assessment of treatment responses across the multicenter sites, to reduce observer bias that might be introduced by the interobserver variability (ie, investigator rating) between the sites and potential bias introduced by the open-label design.

#### Primary Effectiveness Endpoint

The primary effectiveness endpoint was the percentage of participants with the lip volume improved by an LFS ≥ 1 point vs baseline (ie, responders) at Week 6 (Visit 3) after initial treatment, based on the investigator's live assessment. “Responder” was defined as having at least ≥1 grade improvement evaluated with the 5-point validated LFS at a given visit relative to the baseline score (from before treatment). Individual LFS grades for each visit were calculated separately for the upper and the lower lip.

#### Secondary Effectiveness Endpoints

As a secondary endpoint, LFS evaluation was performed at the FU visit, Months 6, 12, and 18. Additionally, as a secondary effectiveness endpoint, aesthetic improvement was evaluated by both the investigator and the patient with the Global Aesthetic Improvement Scale (GAIS), based on comparison with the photographs taken at baseline, when both lips were graded separately (Visit 1, Day 0; Table 1). The FACE-Q questionnaire appearance appraisal scale (U.S. registered trademark of Memorial Sloan-Kettering Cancer Center, 1275 York Avenue, New York, NY 10065) provided another secondary effectiveness endpoint for evaluation of the participant's satisfaction with the lips (Table 2). FACE-Q questionnaires including “Satisfaction with Outcome” and “Satisfaction with Lips” were answered by the participant on a 4-point scale. The upper and lower lip were not graded separately, but overall. For evaluation, the raw scores of the individual items of the FACE-Q questionnaire were added to provide a sum score. This sum score was converted with the respective conversion table of each of the 2 FACE-Q questionnaires, resulting in equivalent Rash transformed scores ranging from 0 (worst) to 100 (best). For both FACE-Q questionnaires, higher scores reflected higher satisfaction. The final secondary effectiveness endpoint was an evaluation of pain with a numerical pain rating scale. Patients were asked to rate their pain perception during the injection procedure. Immediately after injection and 15 minutes later, patients quantified the pain associated with the procedure on an 11-point, semiquantitative numeric pain rating scale from 0 (no pain) to 10 (worst imaginable pain).

**Table 1.** Global Aesthetic Improvement Scale (GAIS) Description

GAIS grade category	GAIS description
Very much improved	Optimal aesthetic result for the implant in this patient.
Much improved	Marked improvement in appearance from the initial condition, but not completely optimal for this patient. A touch-up would slightly improve the result.
Improved	Obvious improvement in appearance from the initial condition, but a touch-up or retreatment is indicated.
No change	The appearance is essentially the same as the original condition.
Worse	The appearance is worse than the original condition.

**Table 2.** FACE-Q Grade Category Description (Satisfaction With Lips)

FACE-Q grade category	Very dissatisfied	Somewhat dissatisfied	Somewhat satisfied	Very satisfied
Shape, lower lip	1	2	3	4
Suit face	1	2	3	4
Smile	1	2	3	4
Full lower lip	1	2	3	4
Style	1	2	3	4
Shape, upper lip	1	2	3	4
Turned up	1	2	3	4
Size	1	2	3	4
Relaxed	1	2	3	4
Full upper lip	1	2	3	4

### Safety Endpoint

The safety endpoint of this clinical study was the occurrence and frequency of any adverse event (AE). All participants of the study were included in the safety analysis set (SAF).

### Statistical Analysis

All primary and secondary endpoints were analyzed descriptively by an independent statistician. The sample size calculation was made for the primary effectiveness endpoint considering the overall population and the subgroups. With a sample size of 100, a 2-sided 95% confidence interval for a single proportion with the large sample normal approximation extended 0.059 in the overall population for an assumed proportion of 0.900 (ie, a responder rate of 90% calculated with nQuery Advanced 8.2.1.0). To cover participants who dropped out or were lost to follow-up, the sample size was increased by 10%, assuring a sufficiently precise estimation of the percentage of responders based on the investigator’s live assessment at Week 6 with the LFS. Paired *t* tests were performed to test the mean change between events at a 5% level of significance.

Furthermore, in a post hoc analysis *P* values were estimated with a mixed model for repeated measure of LFS change from baseline, with baseline LFS scores and touch-up treatment scores as fixed effects, total injection volume as a continuous variate, and repetition factor of lip side (lower or upper).

## RESULTS

### Participant Demographics and Baseline Characteristics

**Figure 3.**
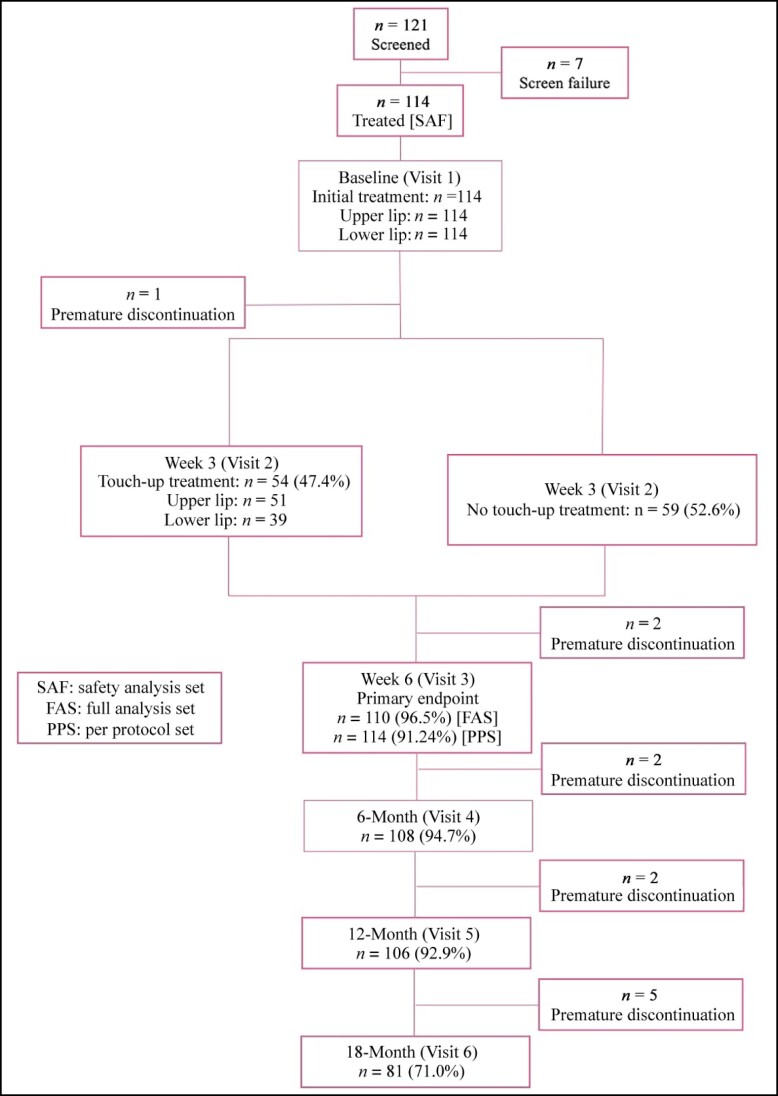
CONSORT flow diagram.

A total of 121 patients signed the consent and were screened for eligibility at 3 study sites (Figure 3). Of these, 114 were eligible, were randomized, and received initial treatment with SLL at the baseline visit. From the eligible group, 47.4% received a touch-up treatment at Week 3; 96.5% attended Week 6 for assessment of the primary endpoint, and 94.7% completed the Month 6 FU. A total of 92.9% of the patients completed the visit at Month 12 (Visit 5) and 71% completed the study at Month 18 (Visit 6). Average patient FU time was 67.46 weeks. Most of the premature discontinuations were for personal reasons or related to the Sars-CoV-2 pandemic, which was ongoing throughout the period of the clinical investigation. In total, 100% of the patients were included in the safety analysis population (SAF, *n* = 114), 110 (96.5%) in the full analysis set (FAS); and 104 (91.2%) in the per protocol set (PPS). Six patients were excluded due to major protocol deviation. The PPS results confirmed the FAS results, and the FAS is described in the graphics and results.

The patients' mean age was 43 years (age range: 18-73); all were females and Caucasian. Of these, 55.3% never smoked, 37.7% were current smokers, and 7.0% were former smokers (Table 3). In relation to the baseline LFS assessment, overall the investigator live assessment and the independent photographic review assessment differed at the baseline visit: the independent photographic reviewer assessment was higher than the investigator live rating: 2.6 for upper lips and 2.7 for lower lips vs 2.1 for upper lips and 2.2 for lower lips, respectively.

**Table 3.** Demographics and Baseline Characteristics of the Safety Analysis Set

Demographic details	Total (safety analysis set) *n* = 114
Age, years	
Mean (range)	43.2 (18-73)
Sex	
Female	114 (100%)
Race	
Caucasian	114 (100%)
Smoking habit	
Current	43 (37.7%)
Former	8 (7.0%)
Never smoked	63 (55.3%)
Investigator, LFS baseline assessment, mean±SD	
Upper lip	2.1 ± 0.77
Lower lip	2.2 ± 0.77
Independent reviewer, LFS baseline assessment, mean±SD	
Upper lip	2.6 ± 0.74
Lower lip	2.7 ± 0.77

Percentages are based on the total number of participants in the specified group of the safety population. A former smoker is defined as greater than 3 months since smoking cessation; a current smoker as less than 3 months since smoking cessation. LFS, lip fullness scale; SD, standard deviation.

### Treatment Administration

On Day 0, a total of 114 patients received SLL; and, at Week 3, 54 (47.4%) patients had a touch-up treatment (Table 4).

**Table 4.** Injection Volume, Technique, and Equipment at Baseline and Week 3 Touch-up Treatment

	Injection volume, mL (mean ± SD)	Injection technique *n* (%)	Injection equipment *n* (%)
Baseline visit, initial treatment, *n* = 114 (100%)			
Upper lip	0.79 ± 0.282	Retrograde, 77 (67.5)	27-gauge 1/2-inch needle, 68 (59.6)
		Bolus, 37 (32.5)	25-gauge × 50-mm cannula, 39 (24.2)
			22-gauge × 70-mm cannula, 7 (6.1)
Lower lip	0.80 ± 0.285	Retrograde, 76 (66.7)	27-gauge 1/2-inch needle, 67 (58.8)
		Bolus, 37 (32.5)	25-gauge × 50-mm cannula, 39 (34.2)
			22-gauge × 70-mm cannula, 7 (6.1)
Both lips	1.59 ± 0.516		
Touch-up treatment, Week 3, *n* = 54 (47.4%)			
Upper lip	0.51 ± 0.3049	Retrograde, 32 (28.1)	27-gauge 1/2-inch needle, 29 (25.4)
		Bolus, 19 (16.7)	25-gauge x 50-mm cannula, 19 (16.7)
			22-gauge × 70-mm cannula, 3 (2.6)
Lower lip	0.50 ± 0.2980	Retrograde, 25 (21.9)	27-gauge 1/2-inch needle, 22 (19.3)
		Bolus, 14 (12.3)	25-gauge × 50-mm cannula, 14 (12.3)
			22-gauge × 70-mm cannula, 3 (2.6)
Both lips	0.85 ± 0.604		
Total volume (both treatments)			
Upper lip	0.97 ± 0.472		
Lower lip	1.042 ± 0.496		
Both lips	2.00 ± 0.915		

Percentages are based on the total number of participants in the specified group of the safety population. SD, standard deviation.

#### Volume Injected

The mean volume of implant gel applied at the initial treatment was ≈0.8 mL per lip and comparable for the upper and lower lips (upper lip: 0.79 mL; lower lip 0.8 mL). In general, for the touch-up treatment a lower volume (≈0.5 mL/lip) was applied than for the initial treatment (upper lip 0.51 mL; lower lip 0.50 mL).

Furthermore, the volume injected depended on the size of the area to be corrected and the desired level of soft tissue augmentation. The mean volume of product applied in both lips at the initial treatment was 1.59 mL, whereas at touch-up treatment the volume was lower (0.85 mL). Overall, the mean total volume of product applied in both lips during both treatments was ≈ 2.0 mL (range: 0.6-4.0).

#### Injection Techniques

Different injection techniques were employed based on the characteristics of the defect under correction and the investigator’s preference. For both initial treatment and touch-up treatment, the most frequent injection technique was the retrograde technique (upper lip 67.5%, lower lip 66.7%). In total, 32.5% of the patients were treated with the bolus technique (upper and lower lips).

#### Injection Devices

A 27-gauge 1/2-inch needle or either a 25-gauge × 50-mm cannula or 22-gauge × 70-mm cannula was utilized to inject the product. At the initial treatment, 27-gauge 1/2-inch needles were employed for both lips in ≈ 60% of patients (upper lip 59.6%, lower lip 58.8%). A total of 34.1% patients were treated with a 25-gauge × 50-mm cannula (upper and lower lips); and 7 patients were treated with 22-gauge × 70-mm cannula (upper and lower lips). At the touch-up treatment, 27-gauge 1/2-inch needles were also preferred for both lips; followed by 25-gauge × 50-mm cannulas and 22-gauge × 70-mm cannulas.

### Effectiveness

#### Primary Effectiveness Endpoint

**Figure 4.**
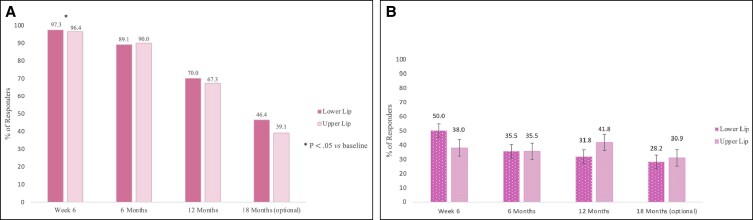
Lip fullness scale (LFS) scores: (A) investigator assessment and (B) independent photographic reviewer assessment.

At Week 6, according to the investigator live assessment, the mean LFS grades of the lower lip increased from 2.1 (slight lip fullness) to 3.5, and the upper lip from 2.0 to 3.3 (PPS, *n* = 104; Table 5). Furthermore, the proportion of responders at Week 6 was 97.3% for the lower lip and 96.4% for the upper lip (Figure 4A). For both lips, the proportion of responders was significantly higher than 90% (lower lip, 95% CI, 92.24-99.43, *P* value = .0071; upper lip, 95% CI, 90.95-99.00, *P* value = .0234).

**Table 5.** Lip Fullness Scale Investigator Live Assessment

Visit (per protocol set, *n* = 104)	Lower lip mean ± SD	Upper lip mean ± SD
Baseline	2.1 ± 0.78	2.0 ± 0.78
Week 6	3.5 ± 0.76	3.3 ± 0.81

Full analysis set, *n* = 114. SD, standard deviation.

The post hoc analysis (Table 6) revealed that the performance of the product measured by LFS score change at Week 6 was influenced by 2 factors: (1) the initial volume deficiency, namely by the LFS score at baseline (*P* = .001); and (2) the total injected volume (*P* = .0002). However, the Week 3 touch-up treatment did not influence the product's performance as evaluated at Week 6.

**Table 6.** Factors Influencing LFS Score Change from Baseline to Week 6 Assessment

Effect	*P* value
Baseline LFS (1, 2, or 3)	.0010
Total injection volume, initial and touch-up treatment	.0002
Touch-up treatment received, yes/no	.9350

Full analysis set, *n* = 114.

**Figure 5.**
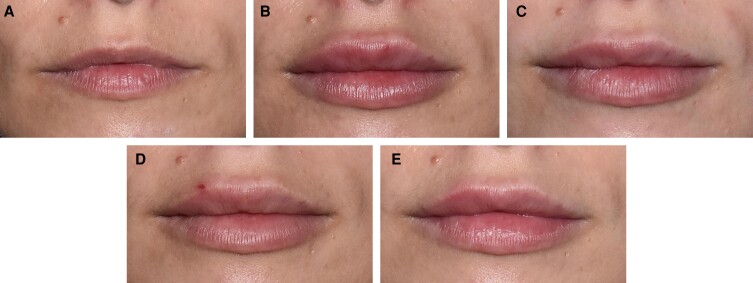
Before and after treatment photographs of a 30-year-old female patient. LFS scale grades assessed by investigator at (A) Day 0: baseline upper lip grade 1, lower lip grade 2; (B) Week 6: upper lip grade 4, lower lip grade 4; (C) Month 6: upper lip grade 4, lower lip grade 4; (D) Month 12: upper lip grade 4, lower lip grade 4; and (E) Month 18: upper lip grade 4, lower lip grade 4. At Day 0, the injected volume for the upper lip was 1.2 mL, and for the lower lip 0.8 mL, with a 27-gauge 1/2-inch needle and bolus technique. At Week 3 during a touch-up treatment the injected volume for the upper lip was 0.4 mL, with a 27-gauge 1/2-inch needle and bolus technique. LFS, lip fullness scale.

**Figure 6.**
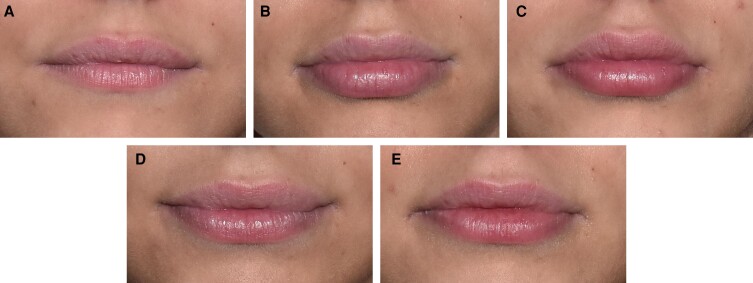
Before and after treatment photographs of a 21-year-old female patient. LFS scale grades assessed by investigator at (A) Day 0: baseline upper lip grade 3, lower lip grade 3; (B) Week 6: upper lip grade 5, lower lip grade 5; (C) Month 6: upper lip grade 4, lower lip grade 4; (D) Month 12: upper lip grade 4, lower lip grade 4; and (E) Month 18: upper lip grade 3, lower lip grade 4. At Day 0 the injected volume for the upper lip was 0.8 mL, and for the lower lip 1.2 mL, with a 27-gauge 1/2-inch needle and bolus technique. At Week 3 during a touch-up treatment the injected volume for the upper lip was 0.7 mL, and for the lower lip 1.3 mL, with a 27-gauge 1/2-inch needle and bolus technique. LFS, lip fullness scale.

**Figure 7.**
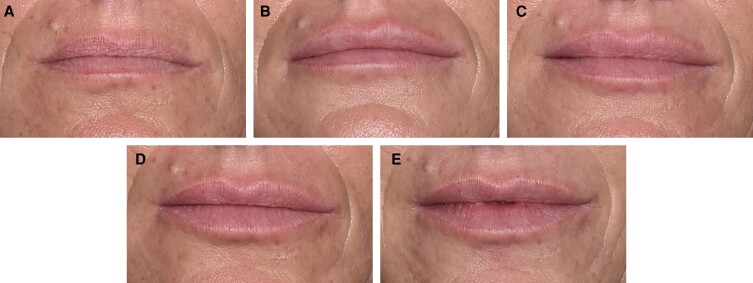
Before and after treatment photographs of a 45-year-old female patient. LFS scale grades assessed by investigator at (A) Day 0: baseline upper lip grade 2, lower lip grade 2; (B) Week 6: upper lip grade 3, lower lip grade 3; (C) Month 6: upper lip grade 3, lower lip grade 3; (D) Month 12: upper lip grade 3, lower lip grade 3; and (E) Month 18: upper lip grade 3, lower lip grade 3. At Day 0 the injected volume for the upper lip was 0.9 mL, and for the lower lip 1.1 mL, with a 27-gauge 1/2-inch needle and bolus technique. At Week 3 during a touch-up treatment the injected volume for the upper lip was 0.3 mL, and for the lower lip 0.1 mL, with a 27-gauge 1/2-inch needle and bolus technique. LFS, lip fullness scale.

Figures 5 to 7 show before and after photographs of 3 patients. LFS scale grades were assessed by the investigator at Day 0, Week 6, Month 6, Month 12, and Month 18.

#### Secondary Effectiveness Endpoints

##### LFS assessment at follow-up

At Month 6, the LFS still showed an improvement of ≥1 grade vs baseline in ≈90% of patients for both lips (Figure 4A). The proportion of responders was 89.1% (95% CI, 81.72-94.2) for the lower lip and 90.0% (95% CI, 82.81-94.9) for the upper lip. At Month 12, there were still ≈70% responders with a slightly higher number of responders for the lower lip (70.0%, 95% CI, 60.52-78.37) than for the upper lip (67.3%, 95% CI, 57.67-75.92); and at Month 18, responders were >40%, with a higher number in the lower lip (46.4%, 95% CI, 36.80-56.12) than the upper lip (39.1%, 95% CI, 29.93-48.86).

With photographs taken at the baseline visit and at each FU, the independent photography reviewer also evaluated the LFS scores. The LFS responder rates resulting from the independent photography reviewer's assessment were lower than the ones assigned by the investigator’s live assessment (Figure 4B).

##### Injection techniques

Responder rates at Week 6 were >90% for both injection techniques (bolus and retrograde) for the upper and lower lips (data not shown). At Month 6, in the bolus technique subgroup the responder rates were >90% for upper and lower lips, but this was not true for the retrograde technique. This difference in LFS responder rates between the injection techniques was not substantial. At Month 12, responder rates were < 90% for both lips, with a substantial difference in LFS responder rates between both techniques for the lower lip in favor of the retrograde technique. At Month 18, responder rates were < 90% and similar for the bolus and retrograde techniques for both lips.

##### Injection equipment

At Week 6, responder rates were >90% for all upper and lower lips; and were similar for 27-gauge 1/2-inch needle and any type of cannula (25-gauge × 50-mm cannula or 22-gauge × 70-mm cannula) (data not shown). After 6 months, in the 27-gauge 1/2-inch needle subgroup responder rates were >90%, but this was not true for any type of cannula. The difference in LFS responder rates between the injection techniques was not substantial. After 12 and 18 months, responder rates were similar, whether the needle or 1 of the cannulas was utilized.

##### Global Aesthetic Improvement Scale

**Figure 8.**
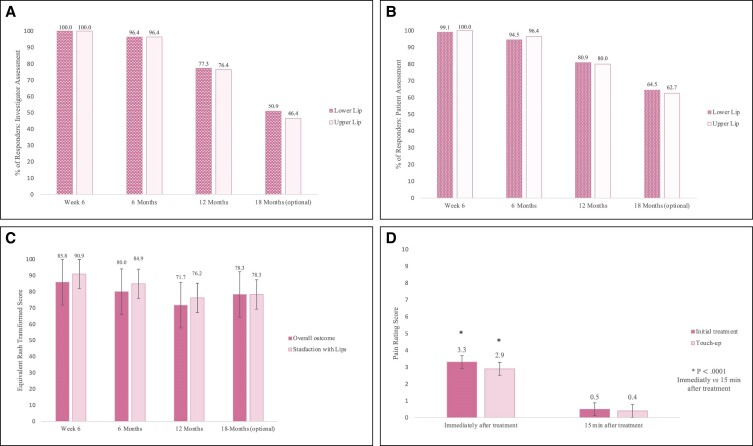
Global aesthetic improvement scale (GAIS) percentage of responders: (A) investigator assessment, and (B) patient assessment. (C) Patient satisfaction by FACE-Q questionnaire. (D) Patient pain perception by numeric pain rating scale.

At Week 6, the investigator’s Global Aesthetic Improvement Scale (GAIS) assessment of aesthetic improvement relative to baseline based on photographs resulted in 100% responders for both lips (upper and lower lips, 95% CI, 96.70-100; Figure 8A). At Month 6, 96.4% of the patients still had an improved aesthetic appearance vs baseline for the lower and upper lips, being considered as responders (upper and lower lips, 95% CI, 90.95-99.00). Only 1.8% of the patients were considered to have no change compared to baseline. The number of aesthetic improvement responders decreased over time. For all time points, assessments of aesthetic improvement of the upper and the lower lips were similar. In general, the patient’s GAIS assessment matched the investigator GAIS evaluation at all time points (Figure 8B).

##### Patient satisfaction

Patient satisfaction with the treatment outcome was high at Week 6 and only slightly decreased over time (FACE-Q satisfaction with outcome, Figure 8C). At Week 6, the mean converted score was 85.8 of 100 points. Furthermore, as described in Supplemental Table 1 (located online at www.aestheticsurgeryjournal.com), 99.1% of patients were pleased with the result at Week 6, 96% at Month 6, and 69.1% at Month 18 (sum of 2 categories “somewhat agree” + “definitely agree”).

Regarding the specific patient satisfaction with the treatment of the lips (FACE-Q satisfaction with lips), patients were also highly satisfied at Week 6, and satisfaction only slightly decreased over time; the mean converted score was 90.9 of 100 points. Furthermore, 92.7% and 67.3% of patients believed that the lips suited their face at Month 6 and Month 18, respectively (sum of 2 categories “somewhat satisfied” + “definitely satisfied”; Supplemental Table 2, located online at www.aestheticsurgeryjournal.com).

##### Numeric pain rating scale

Immediately after the initial treatment, participants rated the pain with a mean pain score of 3.3 (range: 0-8; Figure 8D). Fifteen minutes after treatment, the participants rated the pain considerably lower, with a mean pain score of 0.5 (range: 0-5). This significant reduction of pain was confirmed with a mean difference of −2.81 points between the 2 evaluations (2-sided 95% CI, −3.132-−2.486; *P* value <.0001). The same applied for touch-up treatments, with a mean difference of −2.50 points between initial assessment and assessment after 15 minutes (2-sided 95% CI, −2.851-−2.149; *P* value <.0001).

### Safety: Adverse Events

The safety analysis (Supplemental Tables 3-5, located online at www.aestheticsurgeryjournal.com) was performed on the entire population that received the treatment (SAF, *n* = 114). In total, 92 adverse events (AEs) were documented. For >50% of the patients at least 1 AE was reported. No AE was judged by the investigator to have a relationship to the product; but ≈75% of all AEs (*n* = 142) were assessed as procedure related (ie, injection related). Fewer AEs were reported when with the retrograde technique (77.5%) vs the bolus technique (86.1%). No differences were found in AEs related to the type of equipment (ie, needle vs cannula).

Most AEs were mild or moderate in intensity and resolved within the time frame of the clinical investigation, with no patient discontinuing the clinical study due to an AE. One serious AE (SAE) was reported, which was assessed as not related to the product or to the procedure.

## DISCUSSION

The main objective of this study was to assess the effectiveness and the short- and long-term safety of SLL for lip augmentation in the postmarketing phase. Such postmarket studies are an important method for providing additional efficacy and safety information that goes beyond the scientific data generated in premarket trials. These studies can identify any long-term or rare side effects that were not apparent during premarket trials, providing a more comprehensive safety profile of a product or device. This additional layer of scientific evidence supports healthcare providers in making informed decisions about patient care, including, for example, injection methods. In summary, a total of 114 patients were included and were randomized and monitored for a total period of up to 18 months. The cohort comprised middle-aged Caucasian females with lip volume deficiency and associated LFS scores of 1 to 3. The LFS validated scale allowed evaluation of lip augmentation outcomes after the product injection procedure, a methodology in line with previous studies. Moragas et al stated that a validated scale is the most appropriate way to evaluate lip augmentation outcomes.^11^

The primary effectiveness endpoint of this clinical study was successfully achieved, with a significantly high proportion of responders at Week 6, >90% for both lips. Furthermore, LFS still showed an improvement of ≥1 grade vs baseline for ≈ 90% of patients and for both lips at Month 6. At Month 12, there were still ≈70% responders; and at Month 18, >40% of the patients still had a visible effect. Additionally, according to the investigators, GAIS assessment of aesthetic improvement at Week 6 relative to baseline resulted in 100% responders for both lips. At Month 6, 96.4% of the patients still had an improved aesthetic appearance, with only 1.8% of the patients considered to have no change compared to baseline at this time point. More importantly for the treating physicians, general patient satisfaction with the treatment outcome was high at all time points, with more than 50% still satisfied with the aesthetic outcome after 18 months. Moreover, based on the FACE-Q questionnaire, 96.5% of the participants were satisfied with how nice their lips looked when they smiled at Week 6 after treatment. In our opinion, this supports the above-mentioned hypothesis that low G′ fillers support volume enhancement without distortion of the natural motion of this highly dynamic facial area.^1^ This high level of satisfaction was maintained over time (92.8%, 83.6%, and 67.4% at Months 6, 12, and 18, respectively).

A meta-analysis performed by Czumbel et al found that HA injections effectively increased lip fullness (LF) up to 6 months, with approximately half of the treated participants with significantly increased LF still visible after 12 months.^12^ The results of this clinical study went beyond this previous result, with still ≈ 70% responders at 12 months after the initial treatment. This fact may be due to the improved cross-linking technology of the product, which delays the natural biodegradation of HA.^13,14^

Several factors have been suggested to influence the outcome of lip augmentation with an HA filler like SLL and should be taken into consideration by the injector, such as the injected volume, the number of touch-up treatments, the type of injection technique, the degree of cross-linking in the HA product, its rheological characteristics, the skin type of the patients, the experience of the investigators/treaters, and the evaluation method.^11,12,15-17^ Within the range of rheological characteristics, the elastic modulus, G′, is the most widely reported and perhaps most relevant parameter, because strong correlations have been established between G′ and other parameters such as swelling, the degree of cross-linking, and cohesion.^18^ In this study, the results showed that the outcome of lip augmentation was independent of the touch-up treatment, but dependent on the volume initially injected and the LFS score at baseline. Therefore the product shows a predictable and reliable increase in LF over time. Croma’s proprietary Macro Core Technology allows the manufacturing of such products, with predefined viscoelastic characteristics ensuring product purity. The high molecular weight HA undergoes controlled processing to maintain consistency in the cross-linking process and therefore reproducible product behavior. Multiple filtration steps are complemented by Croma AeroVac purification to reduce air inclusions. Finally, the manufacturing process concludes with a large-scale mixing of cross-linked and non–cross-linked phases leading to a final product with conforming purity, homogeneity, and quality.

As expected, the treatment was safe and well-tolerated, with a mean total volume of ≈2.0 mL of product applied in both lips during both treatments, well below the amount tolerated per year. This shows that even small amounts of injected product produce a high level of satisfaction, not only by physicians but also by the patients themselves. This indicates that with this specific product high volumes of injection may not be necessary to achieve satisfying results.

All AEs during the investigation were in line with comparable study results for similar products currently marketed for this indication. The most frequent AEs reported in the literature are procedural (ie, injection related), such as tenderness, injection site swelling, bruising, injection site masses, and injection site pain, all of which commonly resolve within a few weeks without need for further treatment.^5,12^ This investigation achieved similar results with SLL, in which ≈75% of all AEs assessed were related to the procedure (injection). Importantly, procedural pain was low and transient, with a statistically significant reduction in pain 15 minutes after the procedure, due to the inclusion of lidocaine in the product formulation.

Further analysis showed that fewer AEs were reported with the retrograde technique (77.5%) vs the bolus technique (86.1%). Additionally, no differences were found in AEs related to the type of equipment employed (ie, needle or cannula). As a result, the perception by physicians that a needle is safer than a cannula for HA injection cannot be validated by this study.

Interestingly, the investigator live LFS assessments and the independent photographic reviewer assessments differed at the baseline visit, with the independent photographic reviewer assessments being higher than the investigator live ratings. Raspaldo et al and other groups have reported that live assessment can yield more precise results than photographic analysis based on 3D images, arguing that photographs can modify shadows and smaller rhytids, thereby altering evaluation outcomes.^17,19^ The divergence between the investigator live assessments and the independent photographic reviewer assessments may be explained by the fact that only the investigator performed a live assessment of lip fullness.

### Limitations

Limitations of this study included that all participants were females, and all clinical sites were in Austria, biasing the population treated. As reported by Roman and Zapella, men, Hispanic, and black patients are underrepresented in clinical trials of nonsurgical cosmetic procedures.^20^ Furthermore, as Heidekrueger et al previously reported, the perception of beauty is influenced by an individual's geographic, ethnic, cultural, and demographic background.^21^ Satisfaction and the ideal proportions of LF may have been impacted by this fact. It is known that physicians who practice in Asia, or non-Caucasian physicians, prefer larger lips, while those in Europe and Caucasians prefer smaller lips.^21^ Because countries of residence, ethnic backgrounds, and professions significantly impact individual lip shape preferences, differences in aesthetic preferences can lead to distinctive levels of satisfaction for patients and physicians alike.

The LFS in this study has demonstrated high reliability by expert reviewers and independent testing similar to the Trevidic et al LFS score. However it exhibits lower interrater reliability than the LFS score reported by Carruthers et al.^22,23^

Furthermore, this study was designed without a comparator device control, due to the postmarketing nature of the study. Although efforts were made to define the study population and associated outcomes clearly, we acknowledge the limitations of assessing improvement over time without a randomized control data set with a comparator device.

## CONCLUSIONS

The data presented in this study support the long-lasting effectiveness and safety of SLL, evaluated over a period of up to 18 months. Importantly, patients were highly satisfied with the results throughout the entire time span of the study.

## Supplemental Material

This article contains supplemental material located online at www.aestheticsurgeryjournal.com.

## Supplementary Material

sjae149_Supplementary_Data
