# Supplementary material for: Lip Augmentation With Saypha LIPS Lidocaine: A Postmarket, Prospective, Open-Label, Randomized Clinical Study To Evaluate Its Efficacy and Short- and Long-term Safety
Source: Aesthet Surg J. 2024 Aug 21;45(1):84–97. doi: 10.1093/asj/sjae149 (PMC11634382; doi:10.1093/asj/sjae149)
Supplement: sjae149_Supplementary_Data [file sjae149_supplementary_data.zip › Supplemental Tables 3, 4, 5.docx]

**Supplemental Table 3.** Summary of Adverse Effects (AEs)

|  | Number of AEs | N (%) |
| --- | --- | --- |
| Adverse Event | 192 | 67 (58.77%) |
| Adverse-related to device | 0 | 0 (0%) |
| Adverse-related to procedure | 142 | 63 (55.26%) |
| Serious Adverse Event (SAE) | 1 | 1 (0.88%) |

**Supplemental Table 4**. General Disorders and Administration Site Conditions

|  | Number of AEs | N (%) |
| --- | --- | --- |
| Application Site Pain (Both Lips) | 55 | 47 (41.23%) |
| Injection Site Bruising (Both Lips) | 43 | 33 (28.95%) |
| Injection Site Swelling (Both Lips) | 44 | 34 (29.82%) |

**Supplemental Table 5.** Adverse Effects (AEs)

by Injection Technique and Equipment Used

|  | Bolus  n/N (%) | | Retrograde  n/N (%) | |
| --- | --- | --- | --- | --- |
|  | Needle | Cannula | Needle | Cannula |
| Patients with AEs related to clinical investigation procedure | 31/36 (86.1%) | 0/1  (0.0%) | 7/32 (21.9%) | 25/45 (55.6%) |
| Patients who received Touch-Up treatment | 22/36 (61.1%) | 0/1  (0.0%) | 9/32 (28.1%) | 23/45 (51.1%) |
