# Supplementary material for: Lip Augmentation With Saypha LIPS Lidocaine: A Postmarket, Prospective, Open-Label, Randomized Clinical Study To Evaluate Its Efficacy and Short- and Long-term Safety
Source: Aesthet Surg J. 2024 Aug 21;45(1):84–97. doi: 10.1093/asj/sjae149 (PMC11634382; doi:10.1093/asj/sjae149)
Supplement: sjae149_Supplementary_Data [file sjae149_supplementary_data.zip › Supplemental_Table_1_ASJ-24-0334.docx]

|  | Category | Visit / Total (N=110) n (%) | | | |
| --- | --- | --- | --- | --- | --- |
|  |  | Week 6 | Month 6 | Month 12 | Month 18 |
| Pleased | Definitely disagree | 0 | 0 | 3 (2.7) | 3 (2.7) |
|  | Somewhat disagree | 1 (0.9) | 2 (1.8) | 7 (6.4) | 2 (1.8) |
|  | Somewhat agree | 10 (9.1) | 25 (22.7) | 28 (25.5) | 15 (13.6) |
|  | Definitely agree | 99 (90.0) | 81 (73.6) | 68 (61.8) | 61 (55.5) |
|  | (Missing) | 0 | 2 (1.8) | 4 (3.6) | 29 (26.4) |
| Great | Definitely disagree | 1 (0.9) | 1 (0.9) | 8 (7.3) | 5 (4.5) |
|  | Somewhat disagree | 1 (0.9) | 7 (6.4) | 15 (13.6) | 5 (5.5) |
|  | Somewhat agree | 26 (23.6) | 35 (31.8) | 34 (30.9) | 20 (18.2) |
|  | Definitely agree | 82 (74.5) | 65 (59.1) | 49 (44.5) | 50 (45.5) |
|  | (Missing) | 0 | 2 (1.8) | 4 (3.6) | 29 (26.4) |
| Expected | Definitely disagree | 1 (0.9) | 1 (0.9) | 5 (4.5) | 5 (4.5) |
|  | Somewhat disagree | 2 (1.8) | 6 (5.5) | 11 (10.0) | 2 (1.8) |
|  | Somewhat agree | 26 (23.6) | 28 (25.5) | 32 (29.1) | 17 (15.5) |
|  | Definitely agree | 81 (73.6) | 73 (66.4) | 58 (52.7) | 57 (51.8) |
|  | (Missing) | 0 | 2 (1.8) | 4 (3.6) | 29 (26.4) |
| Look in mirror | Definitely disagree | 1 (0.9) | 1 (0.9) | 4 (3.6) | 4 (3.6) |
|  | Somewhat disagree | 4 (3.6) | 11 (10.0) | 14 (12.7) | 9 (8.2) |
|  | Somewhat agree | 26 (23.6) | 31 (28.2) | 42 (38.2) | 23 (20.9) |
|  | Definitely agree | 79 (71.8) | 65 (59.1) | 46 (41.8) | 45 (40.9) |
|  | (Missing) | 0 | 2 (1.8) | 4 (3.6) | 29 (26.4) |
| Fantastic | Definitely disagree | 2 (1.8) | 1 (0.9) | 9.(8.2) | 5 (5.5) |
|  | Somewhat disagree | 3 (2.7) | 9 (8.2) | 15 (13.6) | 6 (5.5) |
|  | Somewhat agree | 30 (27.3) | 39 (35.5) | 35 (31.8) | 23 (20.9) |
|  | Definitely agree | 75 (68.2) | 59 (53.6) | 47 (42.7) | 46 (41.8) |
|  | (Missing) | 0 | 2 (1.8) | 4 (3.6) | 29 (26.4) |
| Miraculous | Definitely disagree | 2 (1.8) | 2 (1.8) | 9 (8.2) | 7 (6.4) |
|  | Somewhat disagree | 7 (6.4) | 14 (12.7) | 19 (17.3) | 7 (6.4) |
|  | Somewhat agree | 32 (29.1) | 40 (36.4) | 37 (33.6) | 25 (22.7) |
|  | Definitely agree | 69 (62.7) | 52 (47.3) | 41 (37.3) | 42 (28.2) |
|  | (Missing) | 0 | 2 (1.8) | 4 (3.6) | 29 (26.4) |

**Supplemental Table 1**. Summary of Subjects Satisfaction with Overall Outcome, Face-Q^®^ Questionnaire Satisfaction with Outcome (Full Analysis Set). FACE-Q^®^ is a U.S. registered trademark of Memorial Sloan-Kettering Cancer Center, 1275 York Avenue, New York, NY 10065. © 2013 Memorial Sloan-Kettering Cancer Center, Memorial Hospital for Cancer and Allied Diseases, Sloan-Kettering Institute for Cancer Research, Anne Klassen, and Stefan Cano. All rights reserved.
