# Supplementary material for: Lip Augmentation With Saypha LIPS Lidocaine: A Postmarket, Prospective, Open-Label, Randomized Clinical Study To Evaluate Its Efficacy and Short- and Long-term Safety
Source: Aesthet Surg J. 2024 Aug 21;45(1):84–97. doi: 10.1093/asj/sjae149 (PMC11634382; doi:10.1093/asj/sjae149)
Supplement: sjae149_Supplementary_Data [file sjae149_supplementary_data.zip › Supplemental_Table_2_ASJ-24-0334.docx]

|  | Category | Visit / Total (N=110) n(%) | | | |
| --- | --- | --- | --- | --- | --- |
|  |  | Week 6 | Month 6 | Month 12 | Month 18 |
| Shape lower lip | Very Dissatisfied | 0 | 0 | 2 (1.8) | 0 |
|  | Somewhat Dissatisfied | 5 (4.5) | 4 (3.6) | 8 (7.3) | 5 (4.5) |
|  | Somewhat Satisfied | 12 (10.9) | 22 (20.0) | 35 (31.8) | 24 (21.8) |
|  | Very Satisfied | 93 (84.5) | 82 (74.5) | 61 (55.5) | 52 (47.3) |
|  | (Missing) | 0 | 2 (1.8) | 4 (3.6) | 29 (26.4) |
| Suit face | Very Dissatisfied | 0 | 0 | 2 (1.8) | 1 (0.9) |
|  | Somewhat Dissatisfied | 0 | 6 (5.5) | 8 (7.3) | 6 (5.5) |
|  | Somewhat Satisfied | 14 (12.7) | 14 (12.7) | 33 (30.0) | 20 (18.2) |
|  | Very Satisfied | 96 (87.3) | 88 (80.0) | 62 (56.4) | 54 (49.1) |
|  | (Missing) | 0 | 2 (1.8) | 5 (4.5) | 29 (26.4) |
| Smile | Very Dissatisfied | 1 (0.9) | 1 (0.9) | 1 (0.9) | 0 |
|  | Somewhat Dissatisfied | 3 (2.7) | 5 (4.5) | 13 (11.8) | 7 (6.4) |
|  | Somewhat Satisfied | 16 (14.5) | 28 (25.5) | 36 (32.7) | 26 (23.8) |
|  | Very Satisfied | 90 (81.8) | 74 (67.3) | 56 (50.9) | 48 (43.6) |
|  | (Missing) | 0 | 2 (1.8) | 4 (3.6) | 29 (26.4) |
| Full lower lip | Very Dissatisfied | 0 | 0 | 3 (2.7) | 1 (0.9) |
|  | Somewhat Dissatisfied | 4 (3.6) | 5 (4.5) | 11 (10.0) | 13 (11.8) |
|  | Somewhat Satisfied | 20 (18.2) | 26 (23.6) | 33 (30.0) | 16 (14.5) |
|  | Very Satisfied | 86 (78.2) | 77 (70.0) | 59 (53.6) | 51 (46.4) |
|  | (Missing) | 0 | 2 (1.8) | 4 (3.6) | 29 (26.4) |
| Style | Very Dissatisfied | 0 | 0 | 2 (1.8) | 0 |
|  | Somewhat Dissatisfied | 0 | 3 (2.7) | 14 (12.7) | 9 (8.2) |
|  | Somewhat Satisfied | 14 (12.7) | 28 (25.5) | 28 (25.5) | 21 (19.1) |
|  | Very Satisfied | 96 (87.3) | 77 (70.0) | 62 (56.4) | 51 (46.4) |
|  | (Missing) | 0 | 2 (1.8) | 4 (3.6) | 29 (26.4) |
| Shape upper lip | Very Dissatisfied | 1 (0.9) | 0 | 3 (2.7) | 2 (1.8) |
|  | Somewhat Dissatisfied | 1 (0.9) | 13 (11.8) | 17 (15.5) | 8 (7.3) |
|  | Somewhat Satisfied | 23 (20.9) | 26 (23.6) | 39 (35.5) | 32 (29.1) |
|  | Very Satisfied | 85 (77.3) | 69 (62.7) | 47 (42.7) | 39 (35.5) |
|  | (Missing) | 0 | 2 (1.8) | 4 (3.6) | 29 (26.4) |
| Turned up | Very Dissatisfied | 0 | 0 | 4 (3.6) | 0 |
|  | Somewhat Dissatisfied | 1 (0.9) | 9 (8.2) | 11 (10.0) | 13 (11.8) |
|  | Somewhat Satisfied | 24 (21.8) | 29 (26.4) | 35 (31.8) | 28 (25.5) |
|  | Very Satisfied | 85 (77.3) | 70 (63.6) | 56 (50.9) | 40 (36.4) |
|  | (Missing) | 0 | 2 (1.8) | 4 (3.6) | 29 (26.4) |
| Size | Very Dissatisfied | 0 | 0 | 3 (2.7) | 3 (2.7) |
|  | Somewhat Dissatisfied | 2 (1.8) | 11 (10.0) | 14 (12.7) | 9 (8.2) |
|  | Somewhat Satisfied | 23 (20.9) | 30 (27.3) | 41 (37.3) | 27 (24.5) |
|  | Very Satisfied | 85 (77.3) | 67 (60.9) | 48 (43.6) | 42 (38.2) |
|  | (Missing) | 0 | 2 (1.8) | 4 (3.6) | 29 (26.4) |
| Relaxed | Very Dissatisfied | 2 (1.8) | 1 (0.9) | 6 (5.5) | 2 (1.8) |
|  | Somewhat Dissatisfied | 3 (2.7) | 11 (10.0) | 12 (10.9) | 13 (11.8) |
|  | Somewhat Satisfied | 20 (18.2) | 28 (25.5) | 36 (32.7) | 29 (26.4) |
|  | Very Satisfied | 85 (77.3) | 68 (61.8) | 52 (47.3) | 37 (33.6) |
|  | (Missing) | 0 | 2 (1.8) | 4 (3.6) | 29 (26.4) |
| Full upper lip | Very Dissatisfied | 0 | 2 (1.8) | 4 (3.6) | 3 (2.7) |
|  | Somewhat Dissatisfied | 1 (0.9) | 13 (11.8) | 20 (18.2) | 12 (10.9) |
|  | Somewhat Satisfied | 26 (23.6) | 32 (29.1) | 37 (33.6) | 31 (28.2) |
|  | Very Satisfied | 83 (75.5) | 61 (55.5) | 45 (40.9) | 35 (31.8) |
|  | (Missing) | 0 | 2 (1.8) | 4 (3.6) | 29 (26.4) |

**Supplemental Table 2**. Summary of Subjects Satisfaction with Lips, Face-Q^®^ Questionnaire Satisfaction with Lips (Full Analysis Set). FACE-Q^®^ is a U.S. registered trademark of Memorial Sloan-Kettering Cancer Center, 1275 York Avenue, New York, NY 10065. © 2013 Memorial Sloan-Kettering Cancer Center, Memorial Hospital for Cancer and Allied Diseases, Sloan-Kettering Institute for Cancer Research, Anne Klassen, and Stefan Cano. All rights reserved.
